# Self-Regulation of the Fusiform Face Area in Autism Spectrum: A Feasibility Study With Real-Time fMRI Neurofeedback

**DOI:** 10.3389/fnhum.2019.00446

**Published:** 2019-12-20

**Authors:** Jaime A. Pereira, Pradyumna Sepulveda, Mohit Rana, Cristian Montalba, Cristian Tejos, Rafael Torres, Ranganatha Sitaram, Sergio Ruiz

**Affiliations:** ^1^Laboratory for Brain Machine Interfaces and Neuromodulation, Pontifical Catholic University of Chile, Santiago, Chile; ^2^Department of Psychiatry, Faculty of Medicine, Pontifical Catholic University of Chile, Santiago, Chile; ^3^Institute of Cognitive Neuroscience, University College London, London, United Kingdom; ^4^Institute of Medical Psychology and Behavioral Neurobiology, University of Tübingen, Tübingen, Germany; ^5^Biomedical Imaging Center, Faculty of Medicine, Pontifical Catholic University of Chile, Santiago, Chile; ^6^Department of Electrical Engineering, Pontificia Universidad Católica de Chile, Santiago, Chile; ^7^Millennium Nucleus for Cardiovascular Magnetic Resonance, Santiago, Chile; ^8^Institute for Biological and Medical Engineering, Faculty of Engineering, Pontifical Catholic University of Chile, Santiago, Chile

**Keywords:** autism spectrum disorder (ASD), real-time fMRI, neurofeedback, fusiform face area (FFA), facial processing, brain–computer interfaces, neural modulation

## Abstract

One of the most important and early impairments in autism spectrum disorder (ASD) is the abnormal visual processing of human faces. This deficit has been associated with hypoactivation of the fusiform face area (FFA), one of the main hubs of the face-processing network. Neurofeedback based on real-time fMRI (rtfMRI-NF) is a technique that allows the self-regulation of circumscribed brain regions, leading to specific neural modulation and behavioral changes. The aim of the present study was to train participants with ASD to achieve up-regulation of the FFA using rtfMRI-NF, to investigate the neural effects of FFA up-regulation in ASD. For this purpose, three groups of volunteers with normal I.Q. and fluent language were recruited to participate in a rtfMRI-NF protocol of eight training runs in 2 days. Five subjects with ASD participated as part of the experimental group and received contingent feedback to up-regulate bilateral FFA. Two control groups, each one with three participants with typical development (TD), underwent the same protocol: one group with contingent feedback and the other with sham feedback. Whole-brain and functional connectivity analysis using each fusiform gyrus as independent seeds were carried out. The results show that individuals with TD and ASD can achieve FFA up-regulation with contingent feedback. RtfMRI-NF in ASD produced more numerous and stronger short-range connections among brain areas of the ventral visual stream and an absence of the long-range connections to insula and inferior frontal gyrus, as observed in TD subjects. Recruitment of inferior frontal gyrus was observed in both groups during FAA up-regulation. However, insula and caudate nucleus were only recruited in subjects with TD. These results could be explained from a neurodevelopment perspective as a lack of the normal specialization of visual processing areas, and a compensatory mechanism to process visual information of faces. RtfMRI-NF emerges as a potential tool to study visual processing network in ASD, and to explore its clinical potential.

## Introduction

Autism spectrum disorder is a chronic, burdensome (Howlin et al., 2004; Weiss and Lunsky, 2011; Beecham, 2014), highly prevalent neurodevelopmental condition (Kim et al., 2011; Fisch, 2012), and strongly associated with medical and psychiatric comorbidities (Lai et al., 2014). The presentation of ASD is heterogeneous, but is defined by certain clinical characteristics: (1) persistent deficits in communication and social reciprocity, and (2) patterns of repetitive and restrictive behaviors activities and interests, as well as sensorial integration disturbances that are of clinical significance (American Psychiatric Association, 2013). The social, adaptive and mental health prognosis improves with earlier diagnosis and treatment (Fernell et al., 2013). However, there is a subgroup of patients with ASD (patients with fluent language and normal or above normal cognitive capabilities) whose diagnosis usually occurs during late childhood, adolescence, or even in adulthood (Mandell et al., 2005). A better understanding of the neural substrates underlying the core ASD symptoms could help the development of biologically based diagnostic tools to assist clinicians with this challenge (Lai et al., 2014; Varcin and Nelson, 2016).

One of the most important and early impairments in ASD is the abnormal visual processing of human faces (Baron-Cohen et al., 2001a; Calder et al., 2002; Weigelt et al., 2012; Klin et al., 2015). This deficit has been associated with a lack of the typical attentional bias toward social stimuli – mainly faces – that is observed in people with TD from early childhood and throughout life (Dawson et al., 2005; Simion and Giorgio, 2015). There is a deficit in processing visual information emanating from faces (Golarai et al., 2006). This lack of the typical specialization may be compensated by a slower and more cognitively demanding mechanism in ASD individuals with normal or above normal cognitive abilities (Neumann et al., 2006; Clark et al., 2008; Santos et al., 2008; Weigelt et al., 2012; Livingston and Happé, 2017). However, rapid facial processing is necessary for the development of more complex socially relevant cognitive functions (García-Villamisar et al., 2010), such as inter-subjectivity (Yirmiya et al., 1992), pragmatic communication (Mundy et al., 1992) or Theory of Mind (Rogers and Bennetto, 2000; Baron-Cohen et al., 2001a; Calder et al., 2002). All these aspects are usually affected in ASD.

From a neurobiological point of view, progressive structural and functional neural specialization emerges during the visual experience of faces as an important part of typical development (Johnson et al., 2005; Frith, 2007; Adolphs, 2009). This process is characterized by right lateralization (Meng et al., 2012), global integration (Mišić et al., 2014), and local specialization of certain brain areas involved in processing the static, dynamic, emotional and contextual information associated with the visualization of faces (Haxby and Gobbini, 2010). The lateral area of the FG, known as the FFA is considered a critical cortical node for face processing (Ganel et al., 2005; Jiang et al., 2013; Huang et al., 2014) and its hypoactivation is the most consistent finding in research on face processing deficits in ASD (Nickl-Jockschat et al., 2014). Despite the great advances in the description of the cerebral functioning that underlies the deficit of nuclear symptoms of ASD, it has been difficult to translate these findings into useful tools for use both for the diagnostic process in complex clinical settings (e.g., ASD diagnosis on individuals with fluid language and normal IQ) and for new biologically based therapeutic approaches to support the usual current treatment approaches. Therefore, new neuroscientific approaches are needed. Neurofeedback based on real-time fMRI (rtfMRI-NF) is a closed-loop system in which the Blood Oxygenation Level Dependent (BOLD) signal from selected brain regions can be translated to an artificial output that gives contingent information in real-time to the subjects about their brain activity (Shih et al., 2012; Sulzer et al., 2013). RtfMRI-NF has allowed healthy individuals as well as neurological and psychiatric patients to achieve self-regulation of circumscribed brain regions (e.g., Insula, Amygdala, Supplementary Motor Area, FFA), leading to specific neurobiological and behavioral changes (Caria et al., 2007; Zotev et al., 2011; Habes et al., 2016; Paret et al., 2016; Sepulveda et al., 2016). This methodology has been used as a powerful tool to study addiction, schizophrenia, obsessive-compulsive disorder, depression and other brain disorders (Weiskopf et al., 2004; Sitaram et al., 2007, 2017; Weiskopf, 2012; Birbaumer et al., 2013; Ruiz et al., 2014).

The present study is the first one to use rtfMRI-NF for the endogenous neuromodulation of one of the main hubs of face processing, i.e., FFA in autism. In this study, our first aim is to investigate the feasibility of training FFA up-regulation by means of fMRI-NF in people with ASD and to compare this capability with subjects with TD. The neural consequences of FFA up-regulation in both groups will be explored by whole-brain analysis and functional connectivity analysis using both FG as independent seed. In addition to the above aim, the relation between clinical measures and face processing performance (i.e., accuracy of face identity recognition and face emotion recognition) with up-regulation capability will be explored.

## Materials and Methods

### Participants

Eleven male, right-handed volunteers, naïve to real-time fMRI experiments, underwent eight rtfMRI-NF training runs for volitional control of both FFAs. To evaluate the neural effects of this training in TD, six right-handed adult males with TD (i.e., without a clinical history of neurological, psychiatric nor neurodevelopmental disorders nor intellectual disability), and without autistic traits [i.e., negative family history of ASD, scores below 15 in the Social Communication Questionnaire (Rutter et al., 2003), and with Autism Spectrum Quotient (Baron-Cohen et al., 2001b)] below 32 were randomly distributed in two groups (Table 1). Those in the first control group (CG1) participated in a rtfMRI-NF training protocol with contingent information from the FFAs as feedback. The other three individuals participated in a similar protocol, but with non-contingent (sham) feedback (CG2sham).

**TABLE 1:** Demographical information of participants.

	**AG**			**CG1**			**CG2sham**		
			
	***M***	***SD***	**Range**	***M***	***SD***	**Range**	***M***	***SD***	**Range**
AGE (years)	16.52	2.05	14.13–19.50	29.42	4.02	25.67–33.67	35.21	5.24	29.33–39.38
AQ (score)	28.80	5.81	22–34	9.67	6.81	2–15	9.67	4.17	5–13
SCQ (score)	21.80	1.92	19–24	8.33	5.13	4–14	6.33	3.05	3–9

*SCQ, Social Communication Questionnaire; AQ, Autism Spectrum Quotient.*

In a second instance, in order to evaluate the feasibility of this methodology in ASD and its neural effects, five participants with ASD with fluent speech and without intellectual disability participated in a rtfMRI-NF training protocol similar to that carried out by the group CG1. These five participants constituted the “autism group” (AG). Participants in AG were evaluated with a complete clinical battery of tests to establish their clinical profiles and to evaluate if clinical characteristics could predict their performance in the up-regulation of the FFA. Their I. Q. was evaluated with the FIX test, an abbreviated test standardized for the local population that correlates closely with the Wechsler Scale of Intelligence, 4th version (Rosas et al., 2014; Riveros et al., 2015). The ASD diagnosis was made by two independent psychiatrists, based on the DSM 5 criteria (American Psychiatric Association, 2013) and confirmed by two standardized instruments, considered to be gold standard for diagnostic evaluation [the Autism Diagnostic Observational Schedule, ADOS-2 (Lord et al., 2000, 2012), and the ADI-R (Kim et al., 2013)]. The Vineland Adaptive Behavior Scale, second edition (Klin et al., 2007) was used to assess adaptive behavior and social abilities not evaluated in ADOS-2 (Klin et al., 2007) or ADI-R (Lecavalier et al., 2006) (Tables 1, 2).

**TABLE 2:** Clinical information about AG.

	***M***	***SD***	**Range**
FIX^∗^	68.60	14.93	49–91
**ADOS-2 subscales^∗∗^**			
*Communication (C)*	4.00	1.22	3–6
*Social reciprocity (S)*	5.80	0.84	5–7
Total (C + S)	9.80	1.92	8–13
**ADI-R subscales**			
*Social*	19.00	3.74	13–22
*Communication*	13.00	3.74	8–18
*Repetitive behavior*	6.80	2.77	4–11
**Vineland Scale ^∗∗∗^**			
*Chronological Age*	17.76	2.00	15.75–20.5
*Social Age*	15.66	2.45	11.70–17.8
*Social Quotient*	0.88	0.12	0.72–1

*ADOS-2, Autism Diagnostic Observational Schedule second version; ADI-R, Diagnostic Interview Revised; Vineland Scale, Vineland Adaptive Behavior scale, second edition. ^∗^FIX as a percentile for local population (normalized by age); ^∗∗^ADOS-2, module 4 algorithm. ^∗∗∗^Chronological age at Vineland scale performed time.*

Exclusion criteria for all study participants included contraindications for participation in an MRI measurement. After giving a complete description of the study to the participants and to the parents of the adolescents, written informed consent and assent (required in the case of adolescent participants) were obtained. The experimental protocol was approved by the ethics committee of the Pontificia Universidad Católica de Chile.

### Evaluation of Face Processing

To evaluate the relationship between different aspects of facial processing and FFA up-regulation, four tasks were included at the beginning of the first day of rtfMRI-NF training outside the scanner. First, to evaluate memory of faces, the Cambridge Facial Memory Test (CFMT) (Wilson et al., 2010; Hedley et al., 2011) was applied, along with its counterpart, the CCMT (Dennett et al., 2012). Second, to evaluate Theory of Mind and recognition of complex emotions from the eyes, the Reading the Mind in the Eyes task, revised version of “the Eyes task” (Baron-Cohen et al., 2001a) was used. In addition, two tasks were included to evaluate early visual processing of faces, reducing reliance on higher-level cognitive skills: a 4-Alternative Forced Choice task to evaluate the ability to recognize faces (“FIR” task), and a 5-Alternative Forced choice task for the recognition of facial expression of basic emotions (“FER” task). FIR and FER tasks were composed of 32 trials of identical temporal organization (320 s for each task), screened on a 13.3-in. LCD-monitor (Resolution: 1366 × 768; Frame Rate 60 Hz; Viewing distance: 50 cm. app.; PresentationVR 17.1 software, Neurobehavioral Systems, United States). See Supplementary Box for a complete description.

### Real-Time fMRI Training

All subjects participated in 2 days of rtfMRI-NF sessions, with 1 or 2 days of separation between sessions. Each training session began with one localizer run (lasting 4.15 min) to bilaterally localize the FFA to be used as the region of interest (ROI1) from where the BOLD activity was extracted for the next four training runs (each lasting 3.75 min). An anatomical T1 image was acquired at the end of each training day.

#### Functional Localizer

The functional localizer consisted of a block-based paradigm to contrast neutral faces and houses in order to localize left and right FFA (ROI1) (Tong et al., 2000). Four blocks of faces and three blocks of houses were alternating with each other and separated by 21 s of rest between two consecutive blocks (166 volumes; 4.15 min). Each block of houses and faces were composed of 40 images obtained from a pool of 60 images of houses without background and from 60 images of neutral faces obtained from the Karolinska Directed Emotional Faces database (Goeleven et al., 2008) respectively. Each image was presented for 650 milliseconds and separated by 100 milliseconds (black screen) from the next image. A black screen with a white cross in the middle was used for the rest blocks.

The software Turbo Brain Voyager 3.0 (Brain Innovations, Netherlands) was used to select the brain areas of interest that were incorporated into the feedback calculation of the training runs. Feedback information was obtained from ROI1, specifically from the voxels with the greatest activation (Faces > Houses) within the ventral part of each temporal lobe, lateral to the parahippocampal cortices (two cuts of around 5 × 3 voxels each). To cancel the effects of global activation, a transversal slide (9 × 3 voxels) positioned in advance of the third ventricle was used as reference (ROI2).

#### The Real-Time fMRI System

**FIGURE 1:**
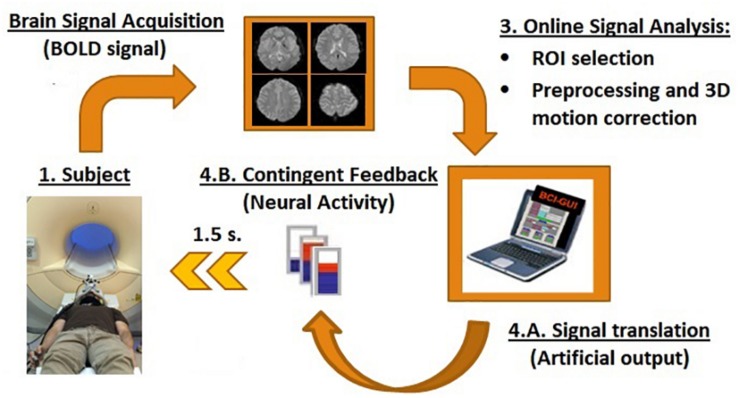
Schematic of the rtfMRI-NF components. RtfMRI-NFs are based on a circular re-entry system in which the BOLD signals of the participants are translated into artificial outputs, i.e., visual contingent feedback such as thermometer with moving bars (refresh time of 1.5 s). It is compound by four main components: **(1)** the participants, **(2)** brain signal acquisition unit, **(3)** signal analysis unit, and **(4)** feedback unit.

An fMRI-NF system similar to those used in previous studies was implemented (Weiskopf et al., 2004; Sitaram et al., 2007; Weiskopf, 2012; Ruiz et al., 2014) (Figure 1). At the beginning of each measurement, participants were positioned in the scanner and reference scans were acquired. Later, using a gradient echo-planar imaging (EPI) sequence (see MR acquisition), functional brain volumes were generated. During image acquisition, brain volumes were transferred in real-time directly from the scanner’s image reconstruction system using the Direct Reconstructor Interface application (Philips Healthcare, Best, Netherlands) to an external computer to analyze it in real-time (Sitaram et al., 2011). A standard personal computer running Turbo Brain Voyager software read the incoming brain volumes to perform real-time 3D motion correction and statistical analysis (Weiskopf et al., 2003). Turbo Brain Voyager parameters were set to match parameters of the EPI acquisition and to obtain the BOLD signal coming from the ROIs at each repetition time (TR: 1.5 s). Custom MATLAB scripts used the signals from the ROIs to compute the feedback by comparing blocks of up-regulation and baseline (Equation 1). The feedback output was stored in a shared text file in the Turbo Brain Voyager computer, which was accessed from the personal computer with PresentationVR 17.1 software (Neurobehavioral Systems, United States). Presentation software read the feedback output file continuously and updated the feedback on the screen at an interval of 1.5 s. The feedback was presented in the form of thermometer bars in an MR compatible visual display system (NordicNeuroLab AS, Norway) (Figure 1).

#### Training Runs and Feedback Calculation

Each participant went through two training sessions. Each training session consisted of four training runs. Each training run started with 10 dummy scans (duration of 15 s) at the beginning of the run to reach the T1 steady state, followed by 4 baseline blocks and 3 up-regulation blocks (each block of 30 s). The dummy scans were later discarded from the analysis. The total duration of training runs was 3 min 45 s. During up-regulation blocks, participants of AG and CG1 groups received contingent visual feedback from their FFAs. Feedback (F) was calculated as:

(1) F=(B⁢O⁢L⁢DU⁢p⁢r⁢e⁢g-B⁢O⁢L⁢DB⁢a⁢s⁢e)R⁢O⁢I⁢ 1

-(B⁢O⁢L⁢DU⁢p⁢r⁢e⁢g-B⁢O⁢L⁢DB⁢a⁢s⁢e)R⁢O⁢I⁢ 2

Where BOLD_*Upreg*_ is the average BOLD signal of a moving window of the last three scans of the up-regulation block, and BOLD_*Base*_ is the average BOLD signal of the preceding baseline block. ROI 1 represented bilateral FFAs selected during the Localizer Run, and ROI 2 was the brain area anterior to the third ventricle which was selected to cancel the effects of global brain activation. Signal artifacts (due to head movement or swallowing) was corrected by replacing any abrupt increases in the BOLD signal by the mean BOLD signal from the preceding time points. Participants of CG2sham were also provided with thermometer feedback but without contingent information, i.e., “sham” feedback (i.e., pseudorandom movement of the thermometer bars).

All participants were instructed to observe the thermometer and to increase the bars. Participants were informed that the movement of the thermometer bars was contingent on the activity of a brain area related to the visual processing of faces. They were also instructed about the 4–6 s delay in the movement of the bar (due to slow hemodynamic response as well as to restrictions imposed by data acquisition and processing). During baseline blocks, a thermometer with stationary bars in the center of the screen was provided to participants, and they were asked to remain at rest (with open eyes) in order to return the BOLD signal to the baseline level.

#### MR Acquisition

The rtfMRI-NF system was implemented using a Philips Achieva 1.5T MR scanner (Philips Healthcare, Best, Netherlands) at the Biomedical Imaging Center of the Pontificia Universidad Católica de Chile. A standard 8-channel head coil was used. For functional image acquisition, we used the Fast Field Echo EPI sequence (TR/TE = 1500/45 ms, matrix size = 64 × 64, flip angle α = 70°, FOV: RL = 210 mm, AP = 210 mm, FH = 79 mm). Sixteen slices (voxel size = 3.2 × 3.3 × 4 mm^3^, gap = 1 mm) were used, oriented with AC/PC alignment to cover the entire temporal and most of the frontal and parietal lobes (Supplementary Figure 1). 150 and 166 scans (10 dummy scans for each one) were performed in each training and functional localizer run respectively. For the superimposition of functional maps on brain anatomy, anatomical T1-weighted brain volumes were acquired, using T1W-3D Turbo Field Echo (magnetization-prepared gradient-echo also known as MPRAGE) sequence (TR/TE = 7.4/3.4 ms, matrix size = 208 × 227, α = 8°, 317 partitions, voxels size = 1.1 × 1.1 × 0.6 mm^3^, TI = 868.7 ms). To prevent discomfort during MRI sessions, pads and air cushions were used to secure the head. Relatives of the ASD participants had the opportunity to accompany the researcher and follow the MRI sessions.

### Offline Brain Imaging Analysis

#### Preprocessing

For brain imaging and ROI analysis, spatial and temporal pre-processing steps were performed with version eight of the SPM (Wellcome Department of Imaging Neuroscience, London, United Kingdom), using 140 functional volumes. The first 10 volumes were discarded to ensure steady-state. Preprocessing included motion correction, realignment, and slice-timing correction. Functional EPI images were co-registered with the acquired T1-weighted image and normalized to Montreal Neurological Institute coordinates. In addition, functional volumes were smoothed with a Gaussian kernel of Full-Width Half Maximum of 8 × 8 × 8 mm.

#### FFA Up-Regulation Calculation

The smoothed and normalized brain volumes were used to evaluate the up-regulation of the BOLD signal separately in the left and right FFAs. The ROI analysis was performed using a sphere of 5 mm^3^ obtained from the left and right parts of ROI1 of each participant (Supplementary Table 1). The magnitude of the left and right FFA (r_FFA_) up-regulation was calculated using the mean BOLD values of regulation and baseline blocks of each run per participant as a percentage as follows:

(2) $$r_{F\,F\,A}=100*\frac{M\,e\,a\,n\,\left(B\,O\,L\,D_{U\,p\,r\,e\,g}\right)-M\,e\,a\,n\,\left(B\,O\,L\,D_{B\,a\,s}\right)}{M\,e\,a\,n\,\left(B\,O\,L\,D_{B\,a\,s}\right)}$$

Where BOLD_*Upreg*_ and BOLD_*Bas*_ represent vectors whose values are extracted from the time-series of regulation and baseline (no feedback) blocks of each training run. The average value of the r_FFA_ during each training session was used as the main measurement of up-regulation performance (one-sample *t*-test compared to zero, *p* two-tailed, 95% confidence). We verified the normality of the data using the D’Agostino and Pearson (omnibus k2) test, and non-parametric tests were used when appropriate.

The “training effect” on up-regulation performance was assessed for each group using two approaches. The difference between the mean r_FFA_ of training session 2 and of training session 1 (Δr_FFA_) was calculated for each subject and then compared against zero by group (one-sample Wilcoxon Signed Rank Test compared with zero, *p* two-tailed, 95% confidence). Second, the slope of the group average of r_FFA_ through the runs, i.e., the “learning slope” of up-regulation was calculated for left and right FFAs (Spearman correlation coefficient, *p* two-tailed, 95% confidence).

#### Variability in FFA Up-Regulation

Given that the variability of the BOLD signal has been associated with neural flexibility and specialization of some brain areas (Nomi et al., 2017), the variability to up-regulate left and right FFAs [as standard deviation (SD) of the BOLD magnitude on each run] was evaluated in the three groups. First, the SD of the r_FFA_ values (SD-r_FFA_) were calculated, and a group comparison (considering all training runs) was carried out for left and right FFA separately (Kruskal–Wallis test, with Dunn’s multiple comparison test as a *post hoc* analysis). Second, the training effect on SD-r_FFA_ values was evaluated for each group, using two approaches. First, the difference between the mean SD-r_FFA_ of session 2 and the mean SD-r_FFA_ of session 1 was calculated for each subject (ΔSD-r_FFA_) and then compared against zero by group (one-sample Wilcoxon Signed Rank Test compared to zero, *p* two-tailed, *p* < 0.05). Second, the slope of the group average of SD-r_FFA_ through the 8 runs or “learning slope” of the variability was calculated for left and right FFA separately (Spearman correlation coefficient, *p* two-tailed, 95% confidence).

### Functional Analysis of the Brain and FFA Up-Regulation

The whole-brain activations and the functional connectivity profile of each FG during FFA up-regulation with rtfMRI-NF were evaluated to obtain a better understanding of the neural networks associated with up-regulation of FFA in ASD.

#### Whole-Brain Analysis

A whole functional brain analysis using all the preprocessed functional images was carried out to evaluate neural activations during the FFA up-regulation guided by the rtfMRI-NF training. A first-level analysis was performed with the SPM. General Linear Modeling was defined considering Regulation and Rest blocks as two independent conditions to map the brain regions recruited. In addition, six generated motion confounds were added to the model and convolution of the regressor with the canonical hemodynamic response function was carried out. A second-level analysis was performed with SPM considering the contrast between regulation blocks and rest blocks (contrast = [Regulation > Rest]) to evaluate specific activations resulting from up-regulation training in each group (one-sample *t*-test per group, *P* < 0.001 and FWE *P* < 0.05; *K* = 10). For the visualization of the brain activations, anatomical automatic labeling or AAL atlas (Tzourio-Mazoyer et al., 2002) and XjView toolbox^1^ were used.

#### Functional Connectivity Analysis

To investigate the network changes during up-regulation, a functional connectivity analysis was carried out. For this purpose, a linear relationship between BOLD activity of different brain regions (AAL atlas) inside of the field of view (Whole brain without the cerebellum and the upper middle part of both parietal and frontal lobes, Supplementary Figure 1) was computed from their correlation coefficients (Friston, 2011) using the CONN toolbox (Whitfield-Gabrieli and Nieto-Castanon, 2012) with left and right FG as the seed regions. FG was chosen as seed given the wide interindividual variability of FFA reported in ASD (Scherf et al., 2010) and replicated in this study (Supplementary Table 1). We used FG as the seed as it encloses our ROI (FFA) due to its greater spatial extent and hence would give us the possibility of making an anatomical and functional comparison between the groups.

Pre-processing consisted of denoising, bandpass-filtering (0.008–0.09 Hz), the inclusion of estimated head motion parameters, use of white matter and cerebrospinal fluid as covariates, linear detrending, and despiking. Bivariate correlations between different brain regions were calculated for regulation blocks taking left and right FGs as seed regions separately. The correlation coefficients between each pair of regions (seed-target) were considered as independent measurement values as follows (Whitfield-Gabrieli and Nieto-Castanon, 2012):

(3) $$r={\left(x^{t}\,x\right)}^{-\frac{1}{2}}\,\left(x^{t}\,y\right)\,{\left(y^{t}\,y\right)}^{-\frac{1}{2}}$$

Where *x* and *y* are vectors of the BOLD time-series for seed ROI and target ROI respectively. To assess similarities and differences of functional connectivity among the participants of different groups, mean pairwise correlation coefficients of functional connectivity (mean z_FC_ values) through all training runs were considered. Results are reported for all significant connections [threshold *P*-FDR (seed corrected) < 0.01, one-sided (positive)] and a group description by lobe is presented.

Group differences in connection strength (mean z_FC_ values) of left and right FGs were evaluated for all brain areas and for the ventral visual stream (i.e., brain areas of the occipital lobe, lingual cortex, FG, parahippocampal cortex, inferior temporal gyrus and ventral area of the temporal pole) (Kravitz et al., 2013; Collins and Olson, 2014) using all significant connections. Such analysis was carried out due: First, based on the importance of the ventral visual stream for specialized visual processing (Kravitz et al., 2013), in particular of faces (Collins and Olson, 2014). Second, due to the particular connection profile described for ASD namely that there are stronger short-range and weaker long-range functional connections (Barttfeld et al., 2011). In particular, higher values of local functional connectivity (Keown et al., 2013), regional activity coherence (Paakki et al., 2010) and degree of centrality (Di Martino et al., 2013) have been found between brain areas of the ventral visual stream in ASD. Both group analyses were performed by one-way ANOVA and the Kruskal–Wallis test, using Tukey’s and Dunn’s multiple comparisons test, respectively, for *post hoc* analysis. All data were checked for normality using D’Agostino and Pearson (omnibus k2) test, and non-parametric tests were used when appropriate. For the visualization, the thickness of the lines connecting the ROIs was represented proportionally to the magnitude of *t* values.

### Autism Spectrum Disorder and Up-Regulation Performance

To evaluate if clinical aspects such as chronological age, IQ, Social Age of Vineland scale, ADOS-2 and ADI-R scores or some aspects of the facial processing performance (i.e., accuracy of FER, FIR, CFMT and Eye-Task and reaction-time of CFMT) are associated with FFA up-regulation performance in subjects with ASD, correlations between these clinical data and r_FFA_ of all training runs were calculated (Spearman correlation coefficient, *p* two-tailed, 95% confidence).

## Results

### Clinical and Facial Processing Profile of Participants

Initially, six participants with ASD were recruited for the study. However, one ASD participant declined to participate in the training due to hearing and tactile discomfort in the scanner. Participants with TD participated in both training days without any sensorial inconvenience. A demographic and clinical summary of the participants can be seen in Tables 1, 2. Although there were no significant group differences in the accuracy achieved in the visual processing tasks (Table 3), participants with TD (the participants in CG1 + CG2sham) showed a faster reaction time in the standardized face memory task (CFMT) compared to the non-social memory task (CCMT) (CFMT Mdn = 3291; CCMT Mdn = 5054; U = 5, *p* = 0.0411, Mann–Whitney *U* test). Participants with ASD showed no such difference (CFMT Mdn = 4436; CCMT Mdn = 4762, *U* = 7, *p* = 0.310, Mann–Whitney *U* test).

**TABLE 3:** Facial processing performance on participants with ASD (AG) and with typical development (participants of CG1 and CG2sham groups).

	**AG**	**CG1 + CG2sham**	
**Task**	**Mdn.**	**Mdn.**	**Statistics**
**FER task**			
*Accuracy*	(0.781)	(0.719)	*U* = 12,*p* = *0.658*
**FIR task**			
*Accuracy*	(0.781)	(0.750)	*U* = *9.5, p* = *0.342*
**CFMT**			
*Accuracy*	(0.667)	(0.688)	*U* = *13, p* = *0.792*
*Reaction Time (msec.)*	(4436)	(3291)	*U* = *7, p* = *0.178*
**CCMT**			
*Accuracy*	(0.736)	(0.757)	*U* = *13.5, p* = *0.833*
*Reaction Time (msec.)*	(4762)	(5054)	*U* = *13, p* = *0.792*
**The Eyes task**			
*Accuracy*	(0.780)	(0.720)	*U* = *9.5, p* = *0.366*

### Training Sessions

All participants underwent eight training runs in two training sessions. Two runs of one participant with ASD had to be discarded from the analysis, as the participant moved his head significantly during the first training run and reported after completing the run that he was using head and eye movements as a strategy to control the thermometer bars. Furthermore, there was a communication loss between the computers due to a temporary hardware problem. In total, 94 training runs (13160 functional images) were used in the analysis.

#### FFA Up-Regulation Magnitude

**FIGURE 2:**
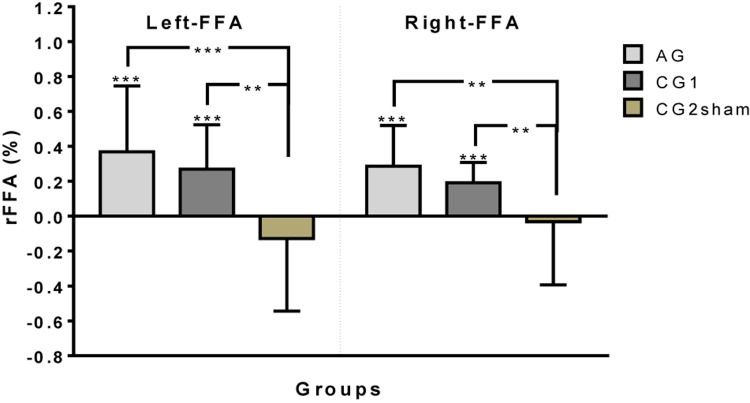
Up-regulation performance (rFFA) by group on left and right FFA (^∗∗^*p* < 0.01, ^∗∗∗^*p* < 0.001).

Both AG and CG1 were able to up-regulate left and right FFAs during all training [left FFA: AG: Mdn = 0.277, *W* = 655, *p* < 0.001; CG1: Mdn = 0.2058, *W* = 292, *p* < 0.001, one-sample Wilcoxon Signed Rank Test compared to zero/right FFA: AG: *M* = 0.286, *t*(37) = 7.55, *p* < 0.001; CG1: *M* = 0.191, *t*(23) = 8.10, *p* < 0.001, one-sample *t*-test, compared to zero]. Moreover, the performance of these groups to up-regulate left and right FFA was significantly high in training session 1 [AG: left FFA: *M* = 0.287, *t*(18) = 3.48, *p* = 0.003; right FFA, Day1: *M* = 0.261, *t*(18) = 5.343, *p* < 0.001/CG1: left FFA: Mdn = 0.226, *W* = 76, *p* < 0.001; right FFA: M = 0.244, *t*(11) = 7.114, *p* < 0.001] and training session 2 [AG: left FFA: Mdn = 0.3546, *W* = 188, *p* < 0.001; right FFA: *M* = 0.311, *t*(18) = 5.297, *p* < 0.001/CG1: left FFA: M = 0.185, *t*(11) = 5.294, *p* < 0.001; right FFA: *M* = 0.139, *t*(11) = 5.406, *p* < 0.001]. In contrast, participants of CG2sham failed to achieve up-regulation in either left or right FFA when both training sessions were taken together (left FFA: Mdn = −0.1417, *W* = −106, *p* = 0.136), or separately [Session 1: left FFA: *M* = −0.131, *t*(11) = 1.553, *p* = 0.149; right FFA: Day1: *M* = −0.0349, *t*(11) = 0.394, *p* = 0.701/Session 2: left FFA: Mdn = −0.1246, *W* = −22, *p* = 0.424; right FFA: *M* = −0.0294, *t*(11) = 0.240, *p* = 0.814] (Figure 2).

Differences between the up-regulation performance of the groups were found on left FFA [H(2)23.35, *p* < 0.001, Kruskal–Wallis test] and right FFA [*F*(2,83) = 11,69, *p* < 0.001, one-way ANOVA]. The *post hoc* analysis for left FFA and right FFA showed better performance in the up-regulation of left and right FFAs in AG and CG1 than in CG2sham (left FFA: AG vs. CG2sham: *p* < 0.001; CG1 vs. CG2sham: *p* = 0.002/right FFA: AG vs. CG2sham: *p* < 0.001; CG1 vs. CG2sham: *p* = 0.008). On the other hand, no differences were found between AG and CG1 (left FFA: *p* > 0.999; right FFA: *p* = 0.332) (Figure 2).

#### FFA Up-Regulation Learning Process

Up-regulation learning in left and right FFAs was evaluated using the following two approaches. First, we evaluated the individual difference in r_FFA_ between session 2 and session 1 (Δr_FFA_) and these values were then compared against zero for each group. Second, the slope obtained from the group mean r_FFA_ of each run (the “activation learning slope”) was estimated. No differences in up-regulating left or right FFA between sessions was found in any group (AG: left Δr_FFA_: Mdn = 0.137, *W* = 13, *p* = 0.125; right Δr_FFA_: Mdn = 0.0691, *W* = 9, *p* = 0.313/CG1: left Δr_FFA_: Mdn = 0.108, *W* = −6, *p* = 0.250; right Δr_FFA_: Mdn = −0.0443, *W* = −6, *p* = 0.250/CG2sham: left Δr_FFA_: Mdn = −0.0150, *W* = 0, *p* > 0.999; right Δr_FFA_: Mdn = −0.0302, *W* = 0, *p* > 0.999). No significant learning slope was found in any group (AG: left FFA: *r*_s_: 0.405, *p* = 0.327; right FFA: *r*_s_ = −0.238, *p* = 0.582/CG1: left FFA: *r*_s_ = −0.619, *p* = 0.115; right FFA: *r*_s_ = −0.476, *p* = 0.2431/CG2sham: left FFA: *r*_s_ = −0.333, *p* = 0.428; right FFA: *r*_s_ = −0.239, *p* = 0.977).

#### FFA Up-Regulation Variability

**FIGURE 3:**
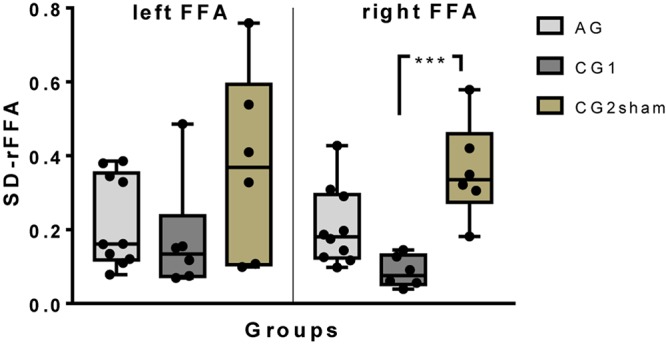
A box-and-whisker plot of the inter-subject variability of up-regulation performance (SD of BOLD magnitude) by group on left and right FFA. All individual results have been plotted (^∗∗∗^*p* ≤ 0.001).

To evaluate whether real feedback contributes to decrease the variability in ROI activity, the BOLD variability was measured during the eight training runs as described in Section “Variability in FFA Up-Regulation.” Group differences between SD-r_FFA_ were found on right FFA but not on left FFA [right FFA: H(2)10.67, *p* = 0.005; left FFA: H(2)5.495, *p* = 0.064]. In a *post hoc* analysis of the right FFA, less variability was found in CG1 than in CG2sham (*p* = 0.004). However, no differences between AG and CG1 (*p* = 0.078), or with CG2sham (*p* > 0.999), were found (Figure 3).

#### Learning Process and Up-Regulation Variability

No differences between ΔSD-r_FFA_ and zero was found for any group (AG: left FFA: Mdn = 0.040, *W* = 9, *p* = 0.313, right FFA: Mdn = −0.0885, *W* = −11, *p* = 0.188; CG1: left FFA: Mdn = 0.0425, *W* = 2, *p* = 0.750, right FFA: Mdn = 0.0663, *W* = 4, *p* = 0.50; CG2sham: left FFA: Mdn = −0.211, *W* = −4, *p* = 0.50, right FFA: Mdn = −0.140, *W* = −4, *p* = 0.50). On analysis of the changes in SD-r_FFA_ during the training runs, a negative correlation between run progression and SD-r_FFA_ was found on left FFA of the CG1 (left FFA: r_s_ = −0.857, *p* = 0.011; right FFA: *r*_s_ = 0.095, *p* = 0.840, ns). No correlation was found between SD-rFFA and run progression on AG or CG2sham (AG: left FFA: *r*_s_ = −0.024, *P* = 0.977, ns; right FFA: *r*_s_ = −0.0714, *P* = 0.882, ns./CG2sham: left FFA: *r*_s_ = 0.048, *P* = 0.935, ns; right FFA: *r*_s_ = 0, *P* > 0.999, ns).

### Functional Analysis of the Brain and FFA Up-Regulation

#### Whole Brain Analysis

**FIGURE 4:**
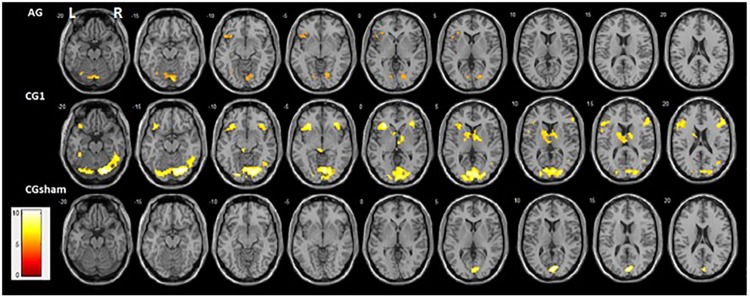
Activation maps of up-regulation of FFAs (Contrast: = up > rest) obtained from whole-brain analysis statistical parametric mapping (SPM) of all runs by group (one-sample *t*-test, *P* < 0.001 and FWE *P* < 0.05; *K* = 10; neurological convention). Bilateral ventral face of the occipitotemporal cortex and bilateral inferior Frontal gyrus activations were found in CG1. In contrast, the left ventral face of the occipitotemporal cortex and left inferior Frontal gyrus activation was found in AG. The right posterior part of the Middle Temporal Gyrus and left Insula were found only in CG1. In addition, cerebellum activation was only present in AG. CG2sham showed only a bilateral Calcarine cortex activation.

Activation profiles in each group (contrast = up > rest; one-sample *t*-test, *P* < 0.001 and FWE *P* < 0.05; *K* = 10) showed clear differences. Although both groups that received contingent feedback (CG1 and AG) showed recruitment of the left FG, left lingual cortex, and left inferior frontal gyrus, recruitment of these brain areas tends to be weaker in AG than in CG1 (Supplementary Table 2). Activations of the right FG, right lingual cortex, right middle temporal gyrus, right inferior frontal gyrus, right insula, and left caudate were found only in CG1. In contrast, bilateral activations of the cerebellum (cerebellum area 6 and vermis area 7) were observed only in AG. The CG2sham group showed activation only in the primary visual cortex (Figure 4). Individual activation maps of the ASD participants are available in the Supplementary Figure 2.

#### Functional Connectivity Analysis

Functional connectivity analysis to evaluate the neural modulation associated with FFA up-regulation in each group was performed using each FGs as two independent seeds (based on the AAL atlas). First, significant functional connections [threshold P-FDR (seed corrected) < 0.01, one-sided (positive)] between the FG and brain areas inside of the field of view were described by lobe for each group. Second, group differences between the functional connectivity strength (mean z_FC_ values) of all significant connections and of connections inside of the ventral visual stream were evaluated taking each FG separately (see section Functional Connectivity Analysis for more information).

**FIGURE 5:**
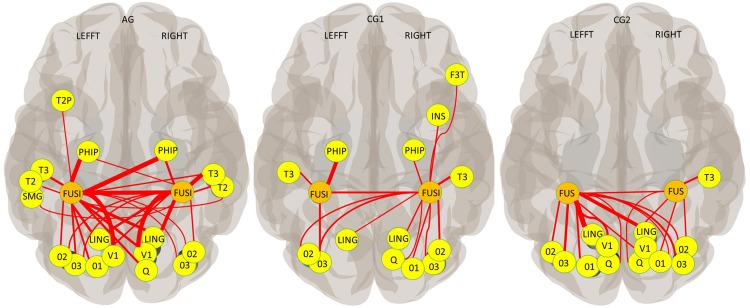
Functional connectivity across all training blocks (AAL Atlas; Seed: each FG; P-FDR (seed corrected) < 0.01; one-sided (positive); thickness proportional to T magnitude). V1 (Calcarine cortex); O1, O2, and O3 (Superior, Middle and Inferior Occipital gyrus, respectively); Q (Cuneus cortex); LING (Lingual cortex); FUSI (FG); SMG (Supramarginal gyrus); T2 and T3 (Middle and Inferior Temporal gyrus); T2P (Temporal Pole, Middle Temporal gyrus); PHIP (Para hippocampus); SMG (Supramarginal gyrus); INS (Insula); F3T (inferior Frontal gyrus, pars triangularis).

Abundant functional connections between the cerebral areas of the occipital lobe (the occipital, lingual and cuneal cortex) and the FG was observed in all three groups, but with interesting differences. GC1 participants showed an ipsilateral and contralateral connection between the occipital brain areas and the right FG, but only ipsilateral connections with the left FG. In contrast, ipsilateral and contralateral connectivities were observed between the occipital lobe and both FGs in AG and CG2sham (Figure 5). Concerning the temporal lobe, although the three groups showed functional connectivity between both FGs, group differences in the connectivity profile were found in this lobe too. Ipsi and contralateral connections between FG and brain areas of the ventral visual stream were observed in AG. In contrast, only ipsilateral connections between the FGs and the inferior Temporal gyrus and parahippocampal cortex were observed in the temporal lobe of CG1. Functional connections to the frontal cortex (ipsilateral connection to right inferior frontal gyrus) and insula (ipsilateral connection to the right insula) were found only in CG1 (Figure 5 and Supplementary Table 3).

No differences were found between groups in the connection strength of the right FG [*F*(2,36) = 3,224, *p* = 0.052, ns]. and of the left FG [*H*(2)0.201, *p* = 0.904, ns] when all brain areas were evaluated. Group differences were found in the connection strength between the right FG and brain areas of the ventral visual stream [*F*(2,33) = 4,81, *p* = 0.015], but no group differences were found in the connection strength of the left FG with these brain areas [*H*(2)0.265, *p* = 0.876, ns]. Specifically, the connections of the right FG and brain areas of the ventral visual stream in AG were stronger than the connections in CG1 (*p* = 0.012). No differences between CG1 and CG2sham (*p* = 0.507, ns), or between AG and CG2sham (*p* = 0.143, ns) were found.

### Autism Clinical Condition and FFA Up-Regulation

The clinical features and facial processing profiles of the patients with ASD were evaluated to explore correlations between them and FFA up-regulation performance to obtain a better understanding of the rtfMRI-NF process in this population (Spearman correlation, two-tailed, 95% confidence interval, *p* < 0.05).

#### Clinical Features and FAA Up-Regulation Performance

Patients with greater symptoms severity of the (ADI-R total score) achieved higher activation values on right FFA during all training runs (right FFA: *r*_s_ = 1, *p* = 0.0167/left FFA: *r*_s_ = 0.6, *p* = 0.0350, ns.) (Supplementary Figure 3). No correlation was found between the up-regulation performance on left and right FFAs and ADOS-2 total score (left FFA: *r*_s_ = −0.667, *p* = 0.267, ns.; right FFA: *r*_s_ = −0.154, *p* = 0.833, ns.), nor Social Communication Questionnaire score (left FFA: *r*_s_ = 0.4, *p* = 0.517 ns.; right FFA: *r*_s_ = 0.7, *p* = 0.233, ns.) nor AQ score (left FFA: *r*_s_ = 0.3, *p* = 0.683 ns.; right FFA: *r*_s_ = 0.4, *p* = 0.517, ns.), nor Social Age of Vineland scale (left FFA: *r*_s_ = −0.8, *p* = 0.133, ns.; right FFA: *r*_s_ = −0.5, *p* = 0.450, ns.), nor chronological Age (left FFA: *r*_s_ = −0.1, *p* = 0.950, ns.; right FFA: *r*_s_ = 0.3, *p* = 0.683, ns.) nor I.Q. (left FFA: *r*_s_ = −0.359, *p* = 0.633, ns.; right FFA: *r*_s_ = −0.154, *p* = 0.833, ns.). No significant relationships between left and right FFA up-regulation performance and facial processing tasks (accuracy of FER, FIR, CFMT and The Eyes task and RT of CFMT) were found (Supplementary Figure 4).

## Discussion

The present study is the first one to our knowledge to examine the application of rtfMRI-NF as a neuroscientific tool by using FFA up-regulation in subjects with ASD (Pereira et al., 2015). In fact, only two rtfMRI-NF studies in autism have been published during this research was carried out. One of them with the aim of up-regulating the posterior part of the superior temporal sulcus (Direito et al., 2019) and the second one looking for strengthening the functional connectivity between that brain region and the inferior parietal lobe (Ramot et al., 2017). In our study, individuals with ASD and TD that received contingent information from the brain region of interest achieved up-regulation of FFA, albeit with considerable differences at neural level.

No differences in the accuracy of face processing tasks were found between TD and ASD subjects, measured before the rtfMRI training. However, TD subjects were faster to answer in face memory tests than in car memory tests. On the other hand, subjects with ASD did not show this advantage for social versus non-social stimuli. This pattern is in line with the development of compensatory mechanisms of high cognitive demand and therefore requiring more processing time (Neumann et al., 2006; Clark et al., 2008; Santos et al., 2008; Weigelt et al., 2012; Livingston and Happé, 2017). These compensatory mechanisms may improve facial processing, but are highly cognitive/emotional wasting and limit real-time social interaction (García-Villamisar et al., 2010).

### FFA Up-Regulation Magnitude

In our study, we show that patients with ASD without intellectual or language deficits were able to upregulate FFA. Individuals with ASD and TD who received real feedback achieved up-regulation of left and right FFAs from the first training session. In contrast, the group that received “sham” feedback (but the same instructions as the other groups) could not up-regulate FFAs in any training session. This suggests that volitional control of the region of interest cannot be learned when there is no contingency between neural activation and the feedback stimulus.

Whether participants with TD or with ASD could show further improvement in their FFA up-regulation performance with a longer training protocol is an open question. Longer protocols may be useful to achieve higher values of FFA up-regulation and translate that learning outside the scanner (Sulzer et al., 2013; Auer et al., 2015). Transferring it to a natural setting could be useful to explore both the association between FFA up-regulation performance and actual face processing improvement, and this method’s potential as an enhancement of usual therapies, as proposed for ASD (Caria and de Falco, 2015) and for other psychiatric disorders (Hanlon et al., 2013; Ruiz et al., 2013b; Buyukturkoglu et al., 2015; Mehler et al., 2018).

### Variability of the FFA Up-Regulation Magnitude

A large variability (SD of the BOLD signal) was found in rFFA values during the training sessions. This concurs with the high intra-subject and inter-subject variability frequently reported in the fMRI literature (Lund et al., 2005; Gaxiola-Valdez and Goodyear, 2012). Despite the small group size in this study, findings on the variability of the BOLD level were significant. In particular, in the case of participants with TD, the contingent neural information contributed to producing less variability in the control of the BOLD activity of both FFAs. There is literature that associates BOLD signal variability with the maturation and specialization process of some brain areas (Nomi et al., 2017), which suggests a possible use of this novel approach to explore the maturation and specialization process (for example of face processing, Meng et al., 2012) in typical and ASD development. However, longer rtfMRI-NF protocols could serve that aim better (Sulzer et al., 2013).

### Functional Analysis of the Brain and FFA Up-Regulation

#### Whole-Brain Analysis

One important finding of the study is the hypoactivation profile of the ventral visual stream – mainly of the right hemisphere – observed in persons with ASD as a consequence of FFA up-regulation guided by real-time fMRI neurofeedback. This finding is in line with the literature showing a specific FG hypoactivation profile when individuals with ASD participate in visual processing tasks with faces as stimuli (i.e., FFA hypoactivation) (Pierce et al., 2004; Nickl-Jockschat et al., 2014) but not with non-social stimuli (Humphreys et al., 2008; Scherf et al., 2010). Moreover, the literature shows that the level of FG activation elicited by non-social stimuli increases with motivational relevance or specialization level, both in the case of individuals with TD (Gauthier et al., 2000; Bilalić et al., 2016; Adamson and Troiani, 2018) and individuals with ASD (Grelotti et al., 2005; Foss-Feig et al., 2016). A possible explanation of FG Hypoactivation observed from the group analysis in participants with ASD may have to do with high variability in the location of FFA obtained in this (Supplementary Table 1) and other studies with participants with ASD (Scherf et al., 2010).

A hypoactivation profile of the inferior frontal gyrus was apparent in participants with ASD unlike those with TD who received contingent information from their FFAs. Inferior frontal gyrus is a brain area associated with the cognitive control network, and with different aspects of volitional cognitive functions (Swick et al., 2008; Della Rosa et al., 2018), as a substrate of the working memory of faces (Courtney et al., 1998; Druzgal and D’Esposito, 2003) and part of the imitation and mirror system (Buccino et al., 2004; Molenberghs et al., 2009). In all these specific cognitive functions individuals with ASD tend to show specific deficits associated with hypoactivation of inferior frontal gyrus (Rogers and Bennetto, 2000; Dapretto et al., 2006; Bookheimer et al., 2008). However, successful neurofeedback training is considered to be disassociated from cognitive effort (Emmert et al., 2016) or any specific mental strategy (Kober et al., 2013). Thus, the inferior frontal gyrus hypoactivation profile shown by participants with ASD could be explained as a specific dysfunction of the facial processing neural network, which could be observable as a consequence of FFA up-regulation. In fact, participants with TD who achieved FFA up-regulation showed a significant ipsilateral functional connection between the FFA and inferior frontal gyrus, but this was absent in ASD individuals and participants in CG2sham (who received the same instructions as CG1 and AG).

In addition, the insula and caudate nucleus were only recruited by those subjects with TD who achieved FFA up-regulation. Both brain areas are considered to be part of the extended network of face processing, in particular of emotional aspects (Haxby and Gobbini, 2010). The insula plays a role in detecting other’s emotions (Thom et al., 2014), in interoception (Critchley et al., 2004) and emotional awareness (Craig, 2009; Gu et al., 2013). In fact, an acquired Insula lesion results in impaired facial recognition of emotion. The caudate nucleus is associated with motivation, reinforcement learning, and reward (Liljeholm and O’Doherty, 2012; Morita et al., 2013; Daniel and Pollmann, 2014; Schultz, 2016; Kasanova et al., 2017). It has been associated, specifically, with social behavior reinforcement (Báez-Mendoza and Schultz, 2013; Bhanji and Delgado, 2014) and giving relative valence to the aspects of faces (Aharon et al., 2001; Lin et al., 2012). A hypoactivation profile of the insula and caudate nucleus have been reported in persons with ASD when performing different social cognition tasks (Pierce et al., 2004; Scott-Van Zeeland et al., 2010; Dichter et al., 2012; Odriozola et al., 2016) which could explain the lack of activation of the insula and caudate nucleus in our participants with ASD. The findings give some insight into the role of the cerebellum. As known, the cerebellum plays a key role in the development and modulation of the motor system (Salman and Tsai, 2016), but also of the higher social cognitive function (Riva, 2000) and reward system (Carta et al., 2019). In typical development, its connections extend to different brain areas, such as the inferior frontal gyrus (Watson et al., 2014), insula (Kaufman et al., 1996; Dobromyslin et al., 2012), ventral tegmental area, and caudate (Fox and Williams, 1970; Carta et al., 2019). In ASD, a disruption of long-range cerebrocerebellar circuits has been reported (Rane et al., 2015). In particular, the lack of correlation between cerebellar activation and caudate activation in ASD may explain the lack of social motivation (Crippa et al., 2016). On the other hand, the recruitment of the cerebellum may be a compensatory mechanism to obtain better social adaptive behavior (D’Mello and Stoodley, 2015; Crippa et al., 2016). This compensatory mechanism seems to be more effective in ASD individuals with fluent language and normal or high I.Q. (Livingston and Happé, 2017; Livingston et al., 2018). Therefore, recruitment of the cerebellum without activation of the striated/caudate nucleus in participants with ASD may be the result of atypical/compensatory development in individuals with ASD.

#### Functional Connectivity Analysis

The up-regulation of FFA in participants with TD resulted in a typical functional connectivity pattern observed previously with different visual tasks aimed at evaluating aspects of the visual processing of faces. Specifically, the connectivity profile in these participants was characterized by connections between the occipital lobe and the right FG, but not with the left FG. The left FG had only an ipsilateral functional connection with the inferior occipital gyrus. This finding can be explained by the typical right lateralization of visual processing of faces (Meng et al., 2012), and by a hierarchical organization of the information (Zhen et al., 2013), whereby the support of the right hemisphere is required to process the representation of faces in the left occipital lobe (faces presented on right visual field) (Verosky and Turk-Browne, 2012). In addition, participants with TD showed significant functional connectivity between the FG and the insula and inferior frontal gyrus as a result of successful FFA up-regulation. As described above, both brain areas have been widely reported to be part of the face-processing network (Haxby and Gobbini, 2010; Zhen et al., 2013). In contrast, participants with ASD showed neither this typical right neural lateralization of functional connectivity in the temporo-occipital cortex, nor functional connectivity between the FG and the insula, or the inferior frontal gyrus. On the other hand, unlike the TD participants, those participants with ASD presented functional connectivity between the FG and areas responsible for higher-order visual processing of faces but of the temporal lobe (the anterior temporal pole and middle temporal gyrus) (Von Der Heide et al., 2013; Zhen et al., 2013; Collins and Olson, 2014).

This atypical functional connectivity pattern could be due to the lack of the normal neural specialization of facial processing (Dawson et al., 2005; Nass and Gazzaniga, 2011), or long-distance brain underconnectivity (Courchesne and Pierce, 2005; Aoki et al., 2013), or it may reflect compensatory/atypical mechanisms for facial processing (Pierce et al., 2001; Joseph et al., 2015). It will have to be explored in future studies with a larger sample and other control groups (e.g., patients with other developmental conditions) if this profile of functional connectivity associated with FFA upregulation is specific to this clinical subpopulation (subjects within the autism spectrum with fluid language and normal IQ), which could bring us closer to obtaining specific diagnostic biomarkers for this clinical subpopulation.

In addition, participants with ASD showed more and stronger connections than individuals with TD between the FG and the brain areas of the ventral visual stream with rtfMRI-NF training. Hyper-connectivity of short-distance connections have been widely reported in ASD (Courchesne and Pierce, 2005; Barttfeld et al., 2011) and have been correlated with symptom severity, social impairment (Supekar et al., 2013; Chien et al., 2015) and savant abilities (Loui et al., 2011).

### Clinical Features and FAA Up-Regulation

This study included ASD subjects without cognitive or language disability for three main reasons. First, because in this protocol participants were required to follow some instructions inside the scanner, which could present difficulty to those with cognitive or language comorbidity. Second, because a more homogenous sample permits a better interpretation of the results. Third, because subjects with ASD who do not have a history of global developmental delay (i.e., with normal IQ and fluent language) are a subgroup in which clinical evaluation is particularly challenging. Hence, being able to evaluate neural differences in this population might contribute to improved diagnostic.

Although one participant was unable to complete the training protocol due to sensory discomfort, the other five participants with ASD finished it without inconvenience. However, given the high prevalence of Sensory Processing Disorder in this population (Marco et al., 2011), a fuller sensory profile evaluation than ADOS-2 and ADI-R seems highly recommended for participants in rtfMRI-NF protocols. Nevertheless, five participants with ASD guided by the rtfMRI-NF managed to up-regulate FFAs and achieve similar activation values to participants with TD who received contingent feedback.

Interestingly, higher values of FFA up-regulation were found in participants with more severe core symptoms of ASD in their childhood (ADI-R total score), despite no correlation was found between the up-regulation performance and the severity of the current core symptoms (evaluated by ADOS-2). Despite of the reduced number of participants (that difficult a generalized explanation), an explanatory hypothesis might be that FFA up-regulatory ability is currently higher in those who developed more communicative and social skills throughout their childhood and adolescence by developing better skills to process information from faces (e.g., individuals with more severe symptoms on their childhood). No correlations between chronological age of ASD participants and up-regulation performance were found (up-regulation performance values were similar through adolescence and adulthood). Moreover, no correlations were apparent between the performance of the different facial processing tasks and FFA up-regulation performance. This can be explained by the absence of differences in the accuracy of these tasks between all individuals as it has been observed in other studies (Harms et al., 2010; Weigelt et al., 2012). Among the study’s limitations is the small size of the sample of participants. However, a strict statistical analysis with correction for multiple comparisons applied during our analyses contributed to the consistency of our analysis. The absence of a group of ASD patients trained with sham feedback could be another potential limitation. However, for ethical considerations we decided, to use instead of a TD control group trained with sham feedback because of two reasons. First, literature shows that up-regulation of a ROI guided by a rtfMRI-NF probably entails behavioral improvement regarding the functions of the respective trained ROI (Sitaram et al., 2007; Linden et al., 2012; Shih et al., 2012; Weiskopf, 2012; Li et al., 2013; Ruiz et al., 2013a; Sulzer et al., 2013; Buyukturkoglu et al., 2015). Second, effective rtfMRI-NF training could bring emotional improvements in the participants due to the experience of successful up-regulation (Mehler et al., 2018). Therefore, it has been preferred to avoid the use of sham feedback in a clinically vulnerable population. Another possible limitation of the study is the difference in age between the groups. Although it was preferred to use subjects with an age (late adolescence or beyond) at which the neural development of face processing is considered done (Pelphrey et al., 2009; de Heering et al., 2012), part of the results of the ASD group may be due to a lag in the neural development of visual processing of faces and not to the ASD condition (Golarai et al., 2010). However, significant results are not expected from the immature TD, thus being better explained by an atypical development (e.g., short-range hyperconnectivity between occipitotemporal brain areas or absence of long-range functional connectivity with insula or inferior frontal gyrus) as previously discussed. Despite that, further studies using this technique to assess changes throughout the development, i.e., at different ages, seems to be useful to obtain a better understanding of the neurodevelopment in ASD. Finally, women were under-represented in the study, a tendency of other studies on ASD (Halladay et al., 2015) despite the quadrupled prevalence of ASD in males compared to females. Behavioral and neural network differences have been found between males and females (Werling and Geschwind, 2013; Coffman et al., 2015; Alaerts et al., 2016), so future use of rtfMRI-NF to explore the facial processing neural network will be useful to study sex differences (Kreiser and White, 2014; Haney, 2016).

## Conclusion and Future Applications

This is the first research to show that the neural networks involved in the visual processing of faces (one of the most important neural substrates affected in ASD) can be studied in participants with ASD using FFA up-regulation with rtfMRI-NF. Consistent differences in facial processing neural networks were found between individuals with typical development and those individuals with ASD. Hypoactivation of the ventral visual stream of the right hemisphere in the participants with ASD and differences in connectivity (with an increase in short but not long connections) in the ASD group compared to the control group with contingent neurofeedback, were some of the main findings. Further studies using this technique are required for the generalization of these findings with studies with a greater number of experimental subjects but also comparing subjects in different stages of development or looking for sex differences. With these limitations, this study demonstrates the technical feasibility of exploring neural differences that may be specific to young adults and adolescents with fluent language and normal IQ who have ASD. In this sense, the research of specific biomarkers for this ASD subpopulation could support the challenging clinical scenario of making the diagnosis of ASD in this population.

## Data Availability Statement

All datasets generated for this study are included in the article/Supplementary Material.

## Ethics Statement

The studies involving human participants were reviewed and approved by the Scientific-Ethical Committee of the Medicine Faculty, Pontificia Universidad Católica de Chile. Written informed consent to participate in this study was provided by the participants’ legal guardian/next of kin and by the participants.

## Author Contributions

JP, SR, and RS conceived and planned the experiments. JP, PS, and CM carried out the experiments. JP, PS, and MR processed the experimental data and performed the analysis. CT and RS verified the analytical methods. JP, SR, and RS contributed to the interpretation of the results. JP took the lead in writing the manuscript. All authors provided critical feedback and helped shape the research, analysis, and manuscript.

## Conflict of Interest

The authors declare that the research was conducted in the absence of any commercial or financial relationships that could be construed as a potential conflict of interest.

## Footnotes

1 http://www.alivelearn.net/xjview

## Abbreviations

AAL: anatomical automatic labeling atlas
ADI-R: the autism diagnostic interview-revised
ADOS-2: autism diagnostic observational schedule version 2
AG: experimental group
ASD: autism spectrum disorder
BOLD: blood oxygenation level-dependent
CCMT: Cambridge car memory test
CFMT: Cambridge face memory test
CG1: control group 1
CG2sham: control group 2
EPI: gradient echo planar imaging
FER: emotion recognition test
FFA: fusiform face area
FG: fusiform gyrus
FIR: face recognition test
ROI: region of interest
rtfMRI-NF: neurofeedback based on real-time functional MRI
SPM: statistical parametric mapping software package
TD: typical neural development.
