# Supplementary material for: Self-Regulation of the Fusiform Face Area in Autism Spectrum: A Feasibility Study With Real-Time fMRI Neurofeedback
Source: Front Hum Neurosci. 2019 Dec 20;13:446. doi: 10.3389/fnhum.2019.00446 (PMC6933482; doi:10.3389/fnhum.2019.00446)
Supplement: Supplementary file 1 [file Data_Sheet_1.pdf]

Supplementary Material

1 Supplementary Figures and Tables

1.1 Supplementary Figures

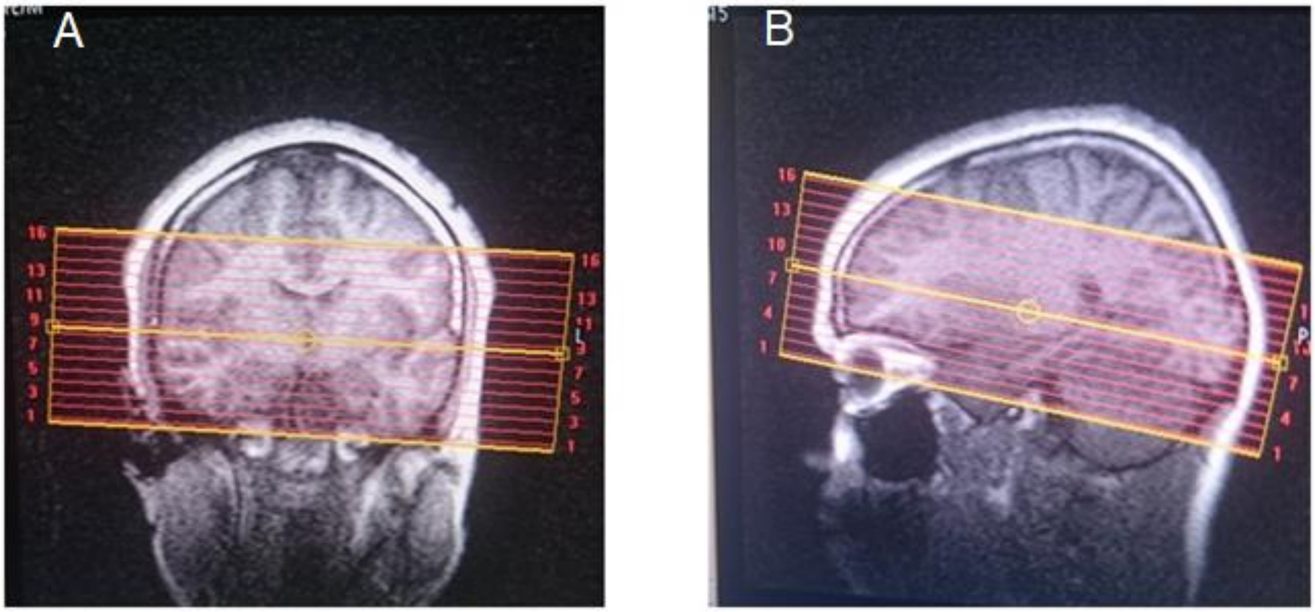

Supplementary Figure 1. Image of the field of view (FOV) used on the fMRI-NF protocol. For the acquisition of functional images, the Fast Field Echo EPI sequence (TR/TE = 1500/45 ms, matrix size = 64 x 64, flip angle α=70°, FOV: RL = 210 mm, AP = 210 mm, FH = 79 mm) was used. Sixteen slices (voxel size = 3.2 x 3.3 x 4 mm^3, gap = 1 mm) were used, oriented with AC/PC alignment to cover the entire temporal and most of the frontal and parietal lobes. The anteroposterior and the lateral views are showed in figures A and B, respectively.

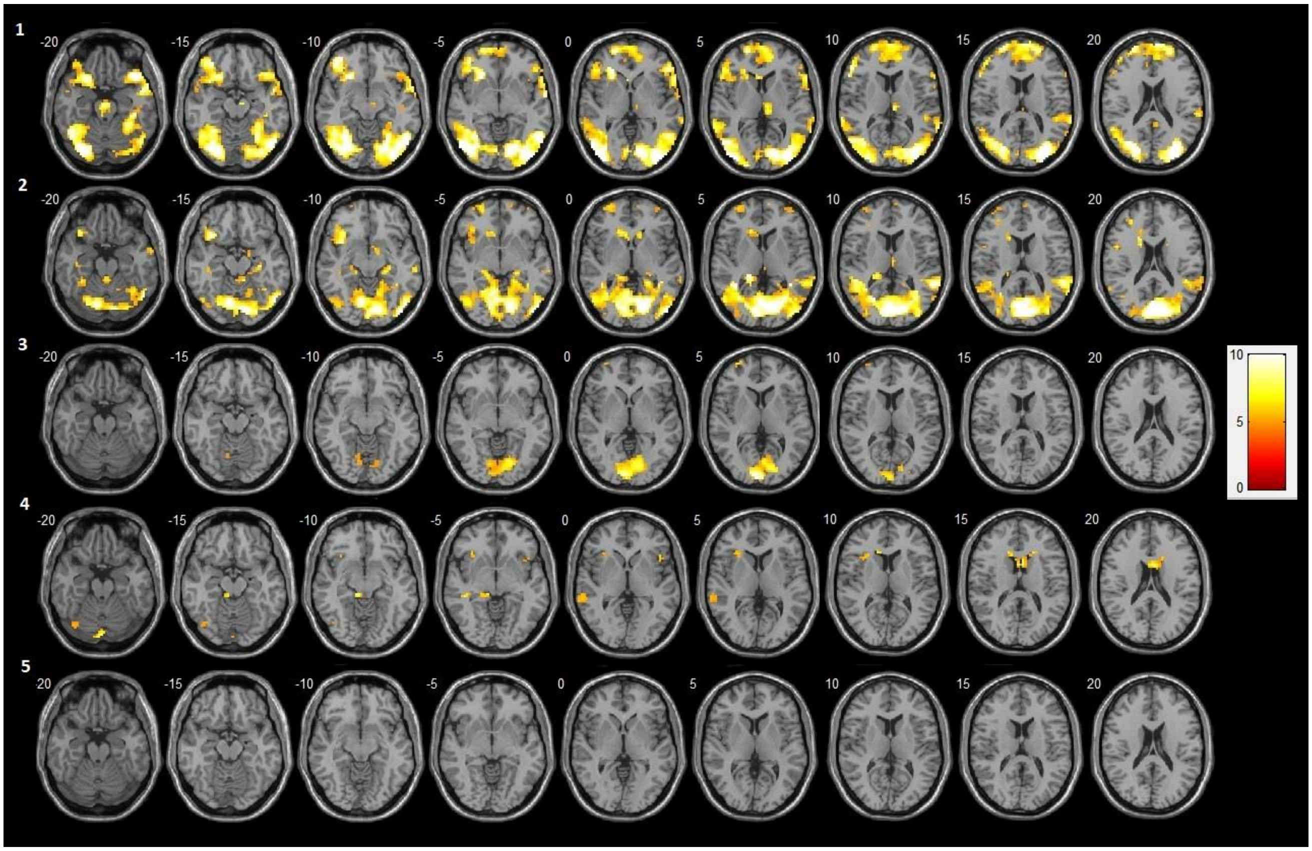

Supplementary Figure 2. Activation maps of FFA up-regulation (Contrast: = up > rest) obtained from whole-brain analysis statistical parametric mapping (SPM) of all runs on each ASD participant (one-sample t-test, P < 0.001 no corrected; K = 5; neurological convention). Eight runs were incorporated into the individual activation maps of participants 1 to 4. Participant 5 completed 6 training runs.

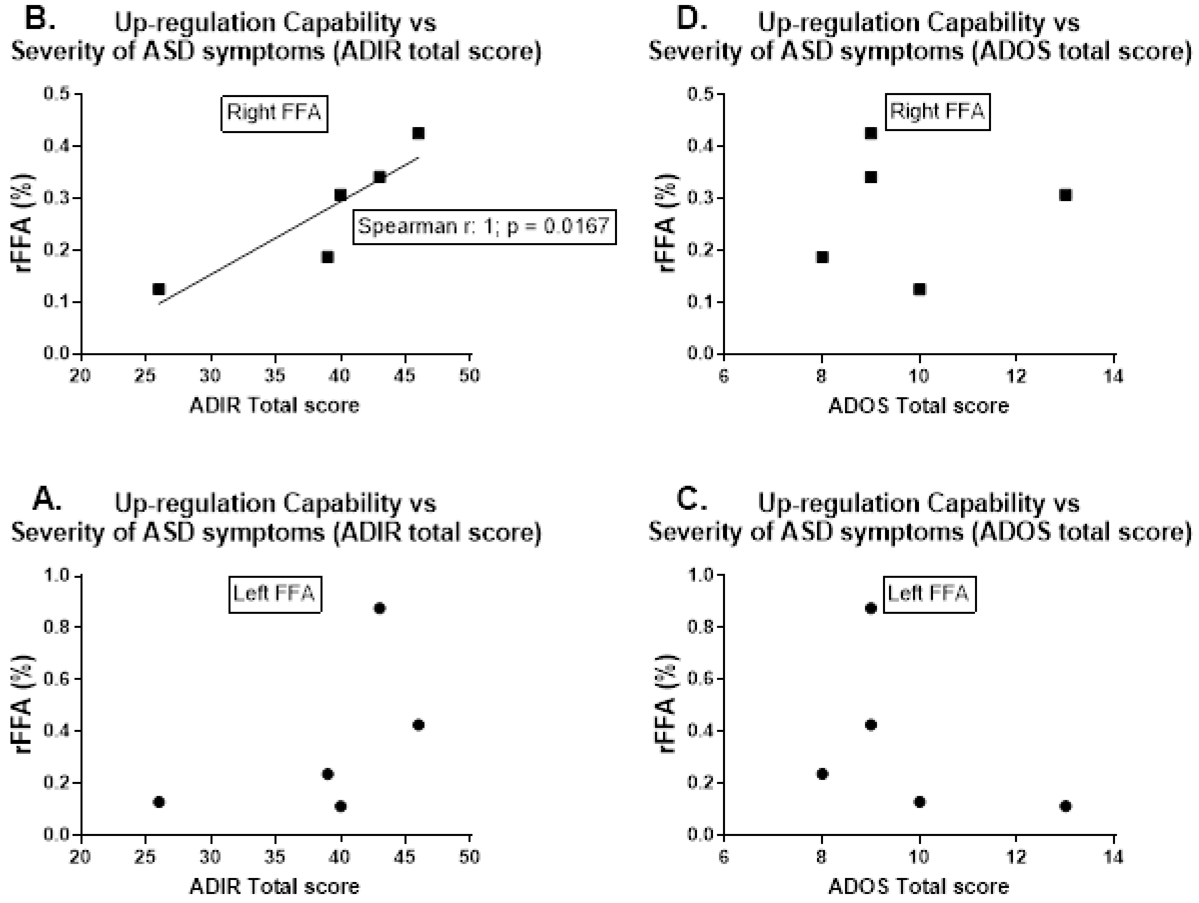

Supplementary Figure 3. Correlations between FFA Up-regulation capability (rFFA) during all training runs and clinical severity (ADIR and ADOS total score) on left FFA (figures A and B respectively), and right FFA (figure C and D). The linear regression was plotted only on figures with significant correlations (Spearman correlation r_s, 95% confidence interval, two-tailed, $p<.05$).

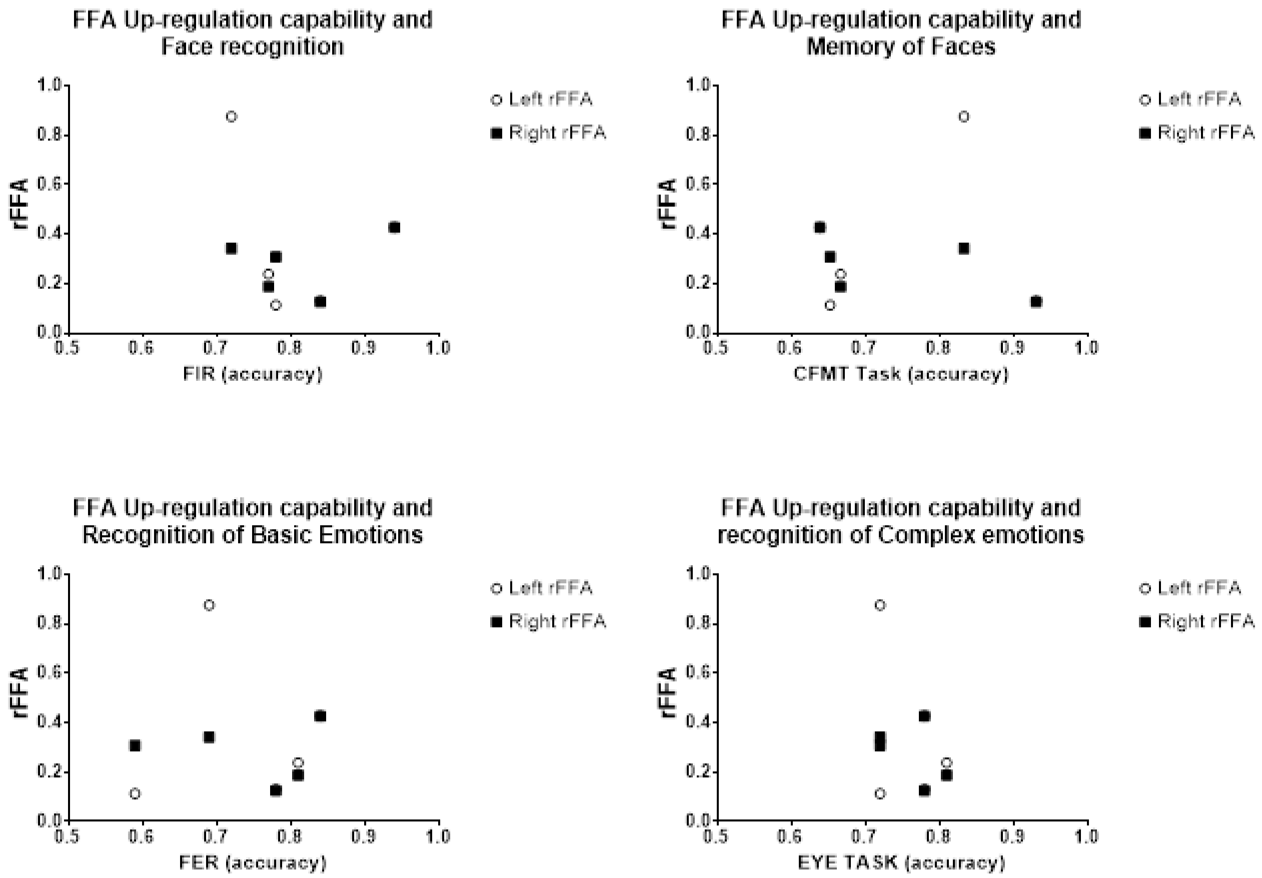

Supplementary Figure 4. Correlations between FFA up-regulation capability during all training runs (rFFA) and facial processing performance (face identification accuracy (measured by FIR), basic emotions (FER), complex emotions trough the eyes (The eye task) and Cambridge Face Memory task (CFMT)). No significant relationships between facial processing and FFA up-regulation capability were found (Spearman correlation, two-tailed, 95% confidence interval, $p<.05$).

1.2 Supplementary Tables

Supplementary Table 1. Region of interest (ROI) localization (MNI) for offline analysis (self-regulation capability). All MNI coordinates are inside of left and right fusiform cortex (AAL atlas).

PARTICIPANT	Day 1	Day 2
AG.1	[-43 -55 -23] ; [48 -58 -22]	[-54 -64 -2] ; [47 -62 0]
AG.2	[-54 -62 0] ; [52 -66 0]	[-51 -58 -10] ; [52 -66 -2]
AG.3	[-39 -67 -19]; [43 -65 -18]	[-39 -67 -19] ; [43 -65 -18]
AG.4	[-56 -56 -10]; [56 -56 -10]	[-56 -56 -10]; [56 -56 -10]
AG.5	[-39 -67 -19]; [43 -65 -18]	[-39 -67 -19]; [43 -65 -18]
CG1.1	[-45 -53 -21];[40 -50 -20]	[-39 -50 -20]; [35 -56 -18]
CG1.2	[-48 -60 -18]; [34 -66 -15]	[-40 -47 -23]; [41 -46 -20]
CG2.3	[-47 -67 -8]; [37 -76 -13]	[-48 -71 -12]; [40 -63 -12]
CG2.1	[-35 -54 -20]; [36 -64 -18]	[-37 -54 -20]; [44 -63 -20]
CG2.2	[-47 -65 -16];[42 -71 -17]	[-50 -68 -13]; [42 -70 -17]
CG2.3	[-39 -67 -19]; [43 -65 -18]	[44 -55 -20]; [-40 -60 -18]

Supplementary Table 2. Significantly activated regions related to self-regulation training (contrast: up-regulation > rest) in the whole brain, univariate analysis, considering the three groups independently (one sample t-test, P < 0.001 & FWE P < 0.05; K = 10).

		peak	peak	cluster	cluster			
Group	AAL region	T	p(FWE-corr)	equivk	p(FWE-corr)	x,y,z {mm}	x,y,z {mm}	x,y,z {mm}
AG								
	Cerebellum_6_R	6,8508	0,000286	98	4,36E-08	7,280004	-79,2	-15
	Calcarine_R	6,0926	0,002275			20,4	-75,92	5
	Vermis_7	5,8632	0,004139			-2,56	-75,92	-20
	Frontal_Inf_Orb_L	5,7737	0,005221	37	3,46E-05	-32,08	22,48	-10
	Frontal_Inf_Orb_L	5,6847	0,006573			-45,2	22,48	-10
	Fusiform_L	5,5197	0,010055			-32,08	29,04	5
	Cerebellum_6_L	5,7160	0,006062	10	0,002421	-28,8	-69,36	-20
	Calcarine_L	5,2525	0,019846	11	0,001982	-5,84	-75,92	5
	Lingual_L	5,1958	0,02289			-12,4	-75,92	0
CG1								
	Lingual_R	12,3487	9,84E-08	728	0	13,84	-82,48	-10

	Fusiform_R	12,1358	1,4E-07			26,96	-79,2	-15
	Lingual_R	10,6954	1,67E-06			7,280004	-79,2	-5
	Insula_L	12,2707	1,12E-07	169	1,11E-16	-28,8	29,04	0
	Frontal_Inf_Orb_L	7,6874	0,000667			-35,36	22,48	-15
	Frontal_Inf_Tri_L	7,34672	0,001416			-51,76	22,48	15
	Frontal_Inf_Orb_R	9,2211	2,73E-05	49	1,43E-08	36,8	29,04	-5
	Insula_R	8,5149	0,000114	53	6,37E-09	43,36	19,2	20
	Frontal_Inf_Tri_R	8,4055	0,000144			49,92	22,48	15
	Frontal_Inf_Tri_R	7,6552	0,000716			56,48	32,32	20
	Fusiform_L	7,8604	0,000458	15	5,41E-05	-35,36	-39,84	-25
	undefined	7,7325	0,000605	150	7,77E-16	17,12	-7,04	5
	Caudate_L	7,5439	0,000914			-15,68	-3,76	20
	L_Thalamus*	7,4661	0,001086			10,56	-0,48	5
	L_Thalamus*	7,3197	0,001504	15	5,41E-05	-2,56	-30	-5
	Temporal_Mid_R	6,5764	0,008211	12	0,00014	40,08	-69,36	20
	Temporal_Mid_R	6,5569	0,008592			46,64	-59,52	10
CG2sham								
	Calcarine_L	11,4064	9,77E-07	66	7,14E-09	0,720004	-85,76	10

Supplementary Table 3. Significant connections during FFA self-regulation blocks (Seed ROIs: each FG; P-FDR (seed corrected) <0.01; one-sided (positive); AAL Atlas).

	Seed: Fusiform L				Seed: Fusiform R			
Group	Targets	T(7)	p-unc	p-FDR	Targets	T(7)	p-unc	p-FDR
AG								
	ParaHippocampal R'	16.98	0.0000	0.0000	Temporal Inf R'	14.91	0.0000	0.0000
	Calcarine L'	15.80	0.0000	0.0000	Fusiform L'	14.89	0.0000	0.0000
	ParaHippocampal L'	15.39	0.0000	0.0000	Lingual R'	12.89	0.0000	0.0000
	Fusiform R'	14.89	0.0000	0.0000	Calcarine R'	10.22	0.0000	0.0002
	Lingual R'	11.24	0.0000	0.0001	Lingual L'	9.16	0.0000	0.0003
	Calcarine R'	9.98	0.0000	0.0001	ParaHippocampal R'	8.83	0.0000	0.0003
	Lingual L'	9.21	0.0000	0.0002	Occipital Inf R'	8.00	0.0000	0.0005
	Cuneus R'	8.73	0.0000	0.0002	Temporal Mid R'	6.35	0.0002	0.0017
	Temporal Inf L'	8.03	0.0000	0.0004	Calcarine L'	6.28	0.0002	0.0017
	Occipital Sup L'	7.26	0.0001	0.0006	Occipital Inf L'	5.34	0.0005	0.0038
	Occipital Inf L'	7.03	0.0001	0.0007	Occipital Mid L'	5.28	0.0006	0.0038
	Occipital Mid L'	5.84	0.0003	0.0019	ParaHippocampal L'	4.99	0.0008	0.0048
	Temporal Inf R'	5.65	0.0004	0.0022	SupraMarginal L'	4.64	0.0012	0.0067
	Occipital Inf R'	4.94	0.0008	0.0044	Temporal Sup R'	4.24	0.0019	0.0100
	Occipital Mid R'	4.48	0.0014	0.0067				
	Temporal Mid R'	4.41	0.0016	0.0067				
	Temporal Mid L'	4.41	0.0016	0.0067				
	Temporal Pol Mid L'	4.36	0.0017	0.0068				
CG1								
	ParaHippocampal L'	13.64	0.0000	0.0001	Temporal Inf R'	8.45	0.0000	0.0019
	Occipital Inf L'	7.97	0.0000	0.0017	Fusiform L'	7.43	0.0001	0.0019
	Fusiform R'	7.43	0.0001	0.0018	Occipital Mid R'	7.35	0.0001	0.0019
	Temporal Inf L'	6.52	0.0002	0.0030	Occipital Inf L'	6.66	0.0001	0.0026
					ParaHippocampal R'	5.65	0.0004	0.0051
					Lingual R'	5.41	0.0005	0.0051
					Occipital Mid L'	5.37	0.0005	0.0051
					Occipital Inf R'	5.31	0.0006	0.0051
					Insula R'	5.04	0.0007	0.0061
					Occipital Sup R'	4.94	0.0008	0.0061
					Lingual L'	4.84	0.0009	0.0062
					Frontal Inf Tri R'	4.55	0.0013	0.0081
					Cuneus R'	4.43	0.0015	0.0085
CG2sham								
	Lingual L'	17.96	0.0000	0.0000	Occipital Inf R'	10.52	0.0000	0.0006
	Lingual R'	12.76	0.0000	0.0001	Occipital Sup L'	8.87	0.0000	0.0007
	Occipital Inf L'	9.37	0.0000	0.0004	Occipital Mid R'	8.44	0.0000	0.0007
	Calcarine L'	8.25	0.0000	0.0007	Lingual R'	8.19	0.0000	0.0007
	Fusiform R'	7.42	0.0001	0.0010	Fusiform L'	7.42	0.0001	0.0011
	Occipital Inf R'	7.23	0.0001	0.0010	Occipital Mid L'	6.51	0.0002	0.0015
	Occipital Sup L'	7.01	0.0001	0.0011	Occipital Sup R'	6.50	0.0002	0.0015
	Occipital Mid L'	6.89	0.0001	0.0011	Temporal Inf R'	6.49	0.0002	0.0015
	Occipital Mid R'	5.55	0.0004	0.0035	Lingual L'	6.19	0.0002	0.0018
	Calcarine R'	5.32	0.0005	0.0040	Calcarine R'	5.16	0.0007	0.0048
	Occipital Sup R'	4.61	0.0012	0.0081	Cuneus L'	4.83	0.0010	0.0063
					Cuneus R'	4.73	0.0011	0.0065

Supplementary Box. The tasks for the rapid recognition of emotions and identity by faces.

The tasks to test the speed of facial emotion recognition and facial identity (FER and FIR task, respectively) begin with a fixation block to ensure that attentional resources are directed to detect the stimuli, followed by a stimulus image (a neutral face for FIR and a face with an expression of negative emotion for FER) presented for 67 milliseconds, i.e., slightly above the identification threshold, but with enough duration to ensure their detection (Calvo and Lundqvist, 2008; Clark et al., 2008; Inuggi et al., 2014; Or and Wilson, 2010). The stimulus presentation was followed by a black screen of 500 milliseconds, followed by the task to be answered (button press) for 5.5 seconds. The FIR task was composed of 3 new neutral faces plus the target (Lai et al., 2012; Serra et al., 2003; Weigelt et al., 2012b). The FER task consisted of a five-choice display with the words “Fear”, “Disgust”, “Anger”, “Sadness” and “Surprise” (Ashwin et al., 2006). **Stimuli**: Faces for the FIR and FER tests were obtained from the Karolinska Directed Emotional Faces (Goeleven et al., 2008). 21 male and 21 female faces (168 images in total) were cropped so that each stimulus fit within an oval window of 15 x 11 cm. and the images of the task fit in an oval of 10 x 7,33 cm. In the FIR task, the stimulus images consisted of 32 neutral faces (sex balanced) and in the FER task, the stimulus images consisted of 32 images of basic emotions of negative valence.
